# C-ME: A 3D Community-Based, Real-Time Collaboration Tool for Scientific Research and Training

**DOI:** 10.1371/journal.pone.0001621

**Published:** 2008-02-20

**Authors:** Anand Kolatkar, Kevin Kennedy, Dan Halabuk, Josh Kunken, Dena Marrinucci, Kelly Bethel, Rodney Guzman, Tim Huckaby, Peter Kuhn

**Affiliations:** 1 Department of Cell Biology, The Scripps Research Institute, La Jolla, California, United States of America; 2 Department of Pathology, Scripps Clinic, La Jolla, California, United States of America; 3 Interknowlogy, Carlsbad, California, United States of America; Northeastern University, United States of America

## Abstract

The need for effective collaboration tools is growing as multidisciplinary proteome-wide projects and distributed research teams become more common. The resulting data is often quite disparate, stored in separate locations, and not contextually related. Collaborative Molecular Modeling Environment (C-ME) is an interactive community-based collaboration system that allows researchers to organize information, visualize data on a two-dimensional (2-D) or three-dimensional (3-D) basis, and share and manage that information with collaborators in real time. C-ME stores the information in industry-standard databases that are immediately accessible by appropriate permission within the computer network directory service or anonymously across the internet through the C-ME application or through a web browser. The system addresses two important aspects of collaboration: context and information management. C-ME allows a researcher to use a 3-D atomic structure model or a 2-D image as a contextual basis on which to attach and share annotations to specific atoms or molecules or to specific regions of a 2-D image. These annotations provide additional information about the atomic structure or image data that can then be evaluated, amended or added to by other project members.

## Introduction

The laboratory environment has changed considerably over the last decade as larger projects and broader scientific challenges have required scientists to cooperate and collaborate with peers around the globe. Thus, few research labs remain isolated, independent units. The internet has enabled these research teams to work in trans-disciplinary environments, with researchers from many disciplines, nations, time zones, and languages working together on large-scale research projects. While this new development enables researchers to address these new challenges, a new approach to conducting daily research is required.

Despite changes to the typical laboratory environment, data often continues to be kept in disparate forms of media. For example, protein structure/activity data annotations and images may be kept in paper lab notebooks, manuscripts might be stored electronically in Portable Document Format (PDF), and molecular structure coordinate files may be stored on a hard disk to be viewed and analyzed in graphical molecular viewers, to name a few. However, most of these storage methods are static, one- or two-dimensional, and not connected in real time and not contextually related. In addition, there is a need to manage the changes in these data over the lifetime of a project and avoid differing versions of documents amongst the project collaborators.

Geographic separation of laboratory units can also present challenges, especially for large-scale research projects. The time and cost for traveling, the efficient distribution of information among collaborators, and the streamlining of workflow processes can be key concerns for laboratory heads and organizational directors.

### Collaboratory Science Systems

To address these concerns, “collaboratory systems” have been put into operation. Collaboratory systems are often computer-based systems that manage research workflow processes and allow different lab units to communicate and share data. Wulf defined a *collaboratory* as a “center without walls, in which the nation's researchers can perform their research without regard to physical location, interacting with colleagues, accessing instrumentation, sharing data and computational resources, and accessing information in digital libraries” (Wulf W (1989) “The national collaboratory.” Towards a National Collaboratory. Unpublished report of a National Science Foundation invitational workshop, New York: Rockefeller University). Bly refines the definition to “a system which combines the interests of the scientific community at large with those of the computer science and engineering community to create integrated, tool-oriented computing and communication systems to support scientific collaborations” [1].

Chin and Lansing state that the research and development of scientific collaboratories has, thus far, been a tool-centric approach [2]. The main goal was to provide tools for shared access and manipulation of specific software systems or scientific instruments. Such an emphasis on tools was necessary in these early development years because of the lack of basic collaboration tools—text chat, synchronous audio, videoconferencing—to support rudimentary levels of communication and interaction. Today, however, such tools are available in off-the-shelf software packages, such as Microsoft LiveMeeting, and IBM Lotus Sametime. The design of collaboratories can move beyond developing general communication mechanisms to evaluating and supporting data sharing in the scientific context. New communication channels can be added to facilitate collaborative exchange of ideas and data. Social network services are an example of a novel communication channel that has attracted large numbers of participants. Services like MySpace and Facebook provide a web site for members with similar interests to interact and share information through a variety of formats, such as, discussion groups, voice and video, file sharing, and email. The success of these services has to be considered within a scientific context to provide an effective on-line environment for scientific collaboration.

A key challenge is to represent and visualize molecular and cellular data as a function of time, space, and ontological state, and then to efficiently and productively manage and share that data with collaborators and the scientific community. Effective proteome-wide prediction of cell signaling interactions require that data on biological relevance, structural accessibility, and molecular sequence be combined to allow for the accurate modeling of cell signaling pathways, for example [3]. When structural genomics intersect with functional proteomics, real-time three-dimensional (3-D) annotation is needed to integrate the two by providing the structural contextual basis upon which to attach the functional information.

Another challenge is to efficiently communicate and discuss these models and their interpretation with collaborators. Research teams are increasingly interdisciplinary and collaborative among laboratories in different departments and institutions located around the world, so it is important for them to have the tools to bridge the gaps of specialization, geography, and time. Researchers must employ data organization and workflow management tools to share the context of their data. The ability to quickly search and retrieve complex 3-D data in real time is critical to the efficiency and productivity of large-scale research work. Currently, the insufficient level of detail made available by published literature, both in print and online, makes it challenging for researchers to share their knowledge. For instance, data from large-scale structural genomics initiatives , such as, PSI I & II (US), SGC (Europe), SPINE (Europe), RIKEN (Japan), will require centralized and automated facilities across continents, new methods to divide labor, and novel tools to disseminate and annotate the data to put it into the larger scientific context of systems biology.

### Available Collaborative Viewing and Annotation Systems

A number of approaches to combine the power of computerized data storage with advanced visualization technology have been developed in the past. Some of the systems below are specific to molecular structures and others are more broad-based client/server collaboration systems offering additional communication and data sharing functionality.

### Kinemage-1992

Kinemage was the first desktop software tool used to visualize macromolecules, making it possible to enhance the communication of 3-D concepts in journal articles with electronic mass distribution of interactive graphics images. Developed at Duke University Medical Center, Kinemage illustrated a particular idea about a 3-D object, rather than neutrally displaying that object [4]. Kinemages, at the time, were a new type of published illustration, providing 3-D views in addition to standard figures, stereo figures, and color plates. Operations on the displayed Kinemage could be rotated in real time, parts of the display could be turned on or off, points could be identified and annotated, and changes between different forms could be animated. This was the first widely available molecular viewing and communication tool that allowed researchers to better communicate ideas that depended on 3-D information.

### Molecular Interactive Collaborative Environment (MICE)-1998

The Molecular Interactive Collaborative Environment (MICE) is an application that provides collaborative, interactive visualization of complex scientific data in 3-D environments within which multiple users can examine complex data sets in real time [5]. MICE is portable and enables users to not only view molecular scenes on their own computer, but to distribute these scenes and interact with other users anywhere on the Internet. Components of MICE are already in use in other San Diego Supercomputer Center projects that require platform-independent collaborative software.

### Biological Collaborative Environment (BioCoRE)-1999

The BioCoRE system developed at the University of Illinois is a large, complex collaboration environment that provides many features, such as data sharing, electronic laboratory notebooks, collaborative molecular viewing and message boards [6]. The BioCoRE system is being used for research and data management as well as for training and uses a combination of a web browser and specialized molecular viewer software to interact with the system and other users.

### EMSL (Environmental Molecular Laboratory Sciences) Collaboratory-2004

The EMSL Collaboratory provides both a Scientific Annotation Middleware (SAM) server and an Electronic Laboratory Notebook (ELN) client as tools for developing collaboration systems [7]. In particular, these tools have been used to develop an NMR Virtual Facility which enables remote NMR operation as well as sharing documents, data, and images and interacting with personnel at the NMR facility.

### iSee-2006

iSee is a software tool that allows users to leverage structural biology data by integrating data from many sources into a single data file and browser [8]. Structures can be annotated with methods, key points of interests, and alignments. Users can then browse the annotated structures, add new annotations, and evaluate existing ones. The files are small enough to be sent as an e-mail message, and the browser runs under Windows, Mac, and Linux operating systems. Though iSee offers saved view states, it is not quite a scientific wiki—where users can make annotations without restrictions—because of its prepackaged annotation system.

### Collaborative Molecular Modeling Environment (C-ME)-Community-based Collaboration Environment-2007

**Figure 1:**
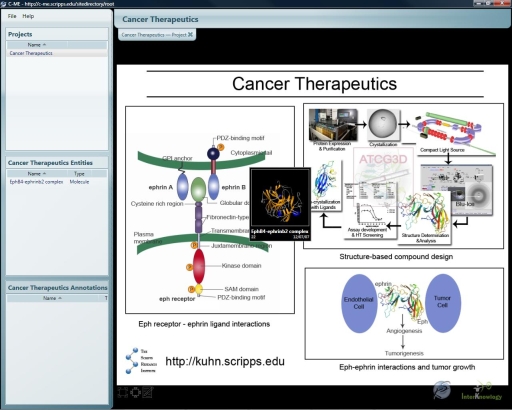
C-ME application window displaying a project. The Project, Entity and Annotations list boxes are displayed along the left hand side and the graphical window for displaying the 3D molecule representations or 2D images to the right. A project can have an associated 2D image to provide additional context as shown in the graphical window for the selected Cancer Therapeutics project. For this project, a protein structure entity is displayed on the project image as a thumbnail image of the cartoon representation of its 3D structure which can then be opened for viewing and annotation by double-clicking it.

Most recently we have developed the Collaborative Molecular Modeling Environment (C-ME), a new collaboratory system that integrates many of the key features available on Kinemage, MICE, iSee, and BioCoRE systems into one thin-client Windows application. The key features that C-ME provides include 2-D image and 3-D molecular structure annotation support in real-time, as well as centralized data storage and organization using hierarchical layers (Projects→Entities→Annotations); the annotation system is not limited to predefined ontological categories (Figure 1).

## Methods

Collaborative Molecular Modeling Environment (C-ME) is a collaboratory system developed for the purpose of supporting research projects involving the molecular systems biology and structural proteomics of the SARS Corona virus as well as a cancer diagnostic research project. These projects require a simple but secure process to store all relevant data in a variety of formats in a single location that are then available either through the C-ME client or through a web interface for those collaborators who do not have the C-ME client. The C-ME backend architecture enables these features and is built on Windows Server 2003, Microsoft SQL Server 2005, and Microsoft Office SharePoint Server 2007 (MOSS 2007). C-ME includes a public domain client application for the Windows XP and Vista operating systems that can be downloaded from http://c-me.scripps.edu.

C-ME is built using commercial off-the-shelf (COTS) software as an open-ended content management and sharing system. The primary advantage for using COTS components to build C-ME is that it reduces overall system development costs and requires less development time—only the components required to perform a specific function need to be developed. Developing a custom system from scratch could involve creating basic functionality, retrieving data from a database, or rendering or moving a three-dimensional image on the screen, all of which might already be available commercially.

C-ME uses COTS components for most of its back-end functions. Windows Server 2003, SQL Server 2005 and MOSS 2007 provide the central authentication, database, and Web infrastructure that enable an application like C-ME to be developed quickly. Windows Server 2003 provides the Active Directory authentication functionality. Users can be added and removed from the directory and permissions assigned according to project or affiliation.

SQL Server 2005 is the relational database management system that provides the data storage, indexing, and retrieval functionality for all data stored on MOSS 2007. Thus, any information exposed by C-ME is actually stored in a SQL Server 2005 database and made available through MOSS 2007 either directly using a browser or through C-ME, using Web services to access the data.

**Figure 2:**
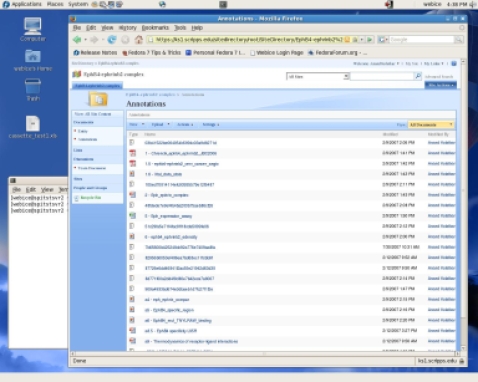
A MOSS 2007 entity site accessed using the Firefox web browser on a Linux computer. All data and annotations for a project or entity are stored in a site on the MOSS 2007 portal. If the C-ME client application is not available, the annotations and data can be accessed via a standard web browser (Mozilla Firefox) as shown here.

MOSS 2007 is the portal system that organizes and makes available the data that is used by C-ME including the root project image, Protein Data Bank (PDB) coordinate files, and 2-D images, as well as all annotations. The user can access this information with C-ME which uses Web services to read and write data to and from the MOSS 2007 server. The user also has the option to access that data through a standard Web browser (Figure 2).

### Software Integration and Implementation Process

C-ME is being developed using a rapid prototyping methodology and new programming tools from Microsoft, including the C# programming language, .NET 3.0 Framework, and the Windows Presentation Foundation (WPF). Other systems, such as Java, Eclipse and NetBeans, also provide built-in features for rapid application development. However, since we are leveraging MOSS 2007 for the data organization and storage functionality, we are using the .NET 3.0 Framework and WPF because of their tight integration with the Microsoft Windows and Server systems [9]. WPF is the graphical subsystem of the .NET 3.0 Framework and provides a programming model for building applications with a separation between the user interface and the underlying logic. The built-in WPF features for rendering 2D and 3D graphic objects were particularly helpful in more quickly assembling the functionality that supports C-ME. The rapid prototyping has enabled us to review iterations of the prototypes in two-week cycles providing bug and usability feedback to the developers for the next cycle of development.

**Figure 3:**
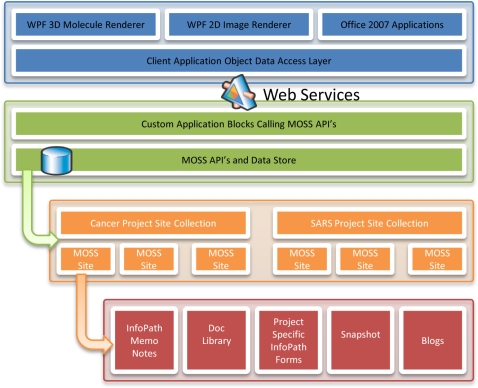
C-ME/MOSS 2007 architecture diagram. Industry standard web services (triangle) are the links between the C-ME client application (top–blue) and the research project information stored in databases that are accessed through the Microsoft Office SharePoint Server 2007 API (middle–green). The data and annotations used in C-ME reside in various MOSS 2007 portal project and entity web sites (bottom–orange). Each project and entity has its own site for data and annotations (bottom–red).

**Figure 4:**
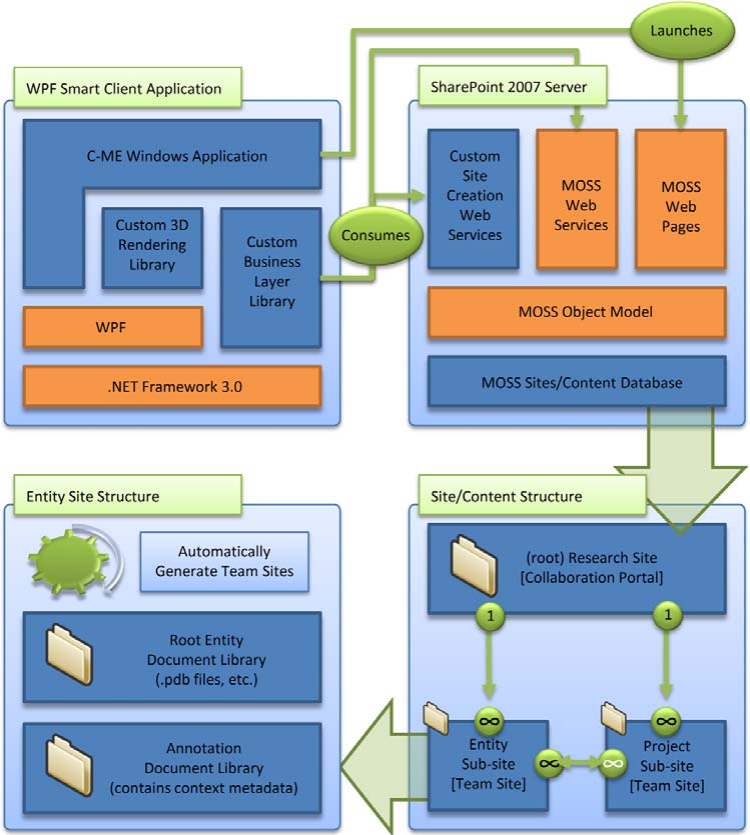
Data flow from the C-ME client to the MOSS 2007 portal. Information entered into C-ME flows through the system to the appropriate levels in the hierarchy, which is managed by the MOSS 2007 data management component. The C-ME client (upper left) reads and writes data to and from MOSS 2007 (upper right). MOSS 2007 organizes C-ME data and annotations into a research site with multiple project sites (lower right). Each of the project sites can contain multiple entity sites (lower left) for each 3D molecule or 2D image to be annotated and shared.

Figure 3 illustrates the relationships among the image rendering components and the back-end components, which communicate through industry-standard web services. The C-ME graphical client application communicates via web service calls to the MOSS 2007 application programming interface (API) to read or write data to a specific MOSS 2007 entity or project site or to create new project and entity sites. Figure 4 shows the flow of data as it is entered into C-ME and directed to the appropriate site on the MOSS 2007 server which uses SQL Server 2005 as the relational database system for data storage.

## Results and Discussion

### C-ME as a 3D Community-Based Collaboratory System

Allowing multiple participants to jointly create and edit web pages is known as a “wiki,” first developed and implemented by Ward Cunningham in 1995 (http://en.wikipedia.org/wiki/Wiki). The Wikipedia is perhaps the most widely known and used wiki and is essentially an encyclopedia collaboratively written by volunteers [10], [11].

By allowing researchers to publish their data sets to a larger community through a publicly or privately available data portal, C-ME creates the possibility of a “wiki-like” research community, where any number of authorized participants can view and annotate research results interactively in a specific 2-D or 3-D context. Importantly, C-ME is a collaboratory system that enables the sharing and analysis of biological data with project level security in addition to the large wiki features through:

Controlled access–Users are authenticated against an Active Directory to determine their level of access to projects and entities. Access can also be provided to accounts residing on external LDAP repositories via MOSS. Anonymous read-only access is also available.

Simple graphical interface–The graphical interface provides a standard set of mouse and keyboard controls to manipulate and annotate images and structures.

Data organization–Data entered into C-ME is organized in a hierarchical system that allows creation of projects containing multiple sub-projects.

Data consistency–All data is stored in one location so that all users access the same data from the C-ME application. All changes are immediately available to all users.

Robust data backend–All C-ME data is stored in an industry-standard SQL Server 2005 database accessed through the MOSS 2007 application programming interface.

Context-specific annotation–Annotations are related graphically to the feature in the image or molecular structure that is pertinent.

The system supports several data formats, has data translation capabilities, and is able to interact and exchange data with other sources, such as external databases. By leveraging MOSS 2007 functionality, C-ME offers subscription capabilities, performs user authentication, establishes and manages permissions and privileges, and has data encryption capabilities to ensure secure data transmission as part of its security package.

### Data Management & Information Hierarchies

C-ME organizes data into a hierarchical structure that displays the organization of a data set at three abstraction levels: the Project (multiple) level, the Entity level, and the Annotation level. Each layer of the hierarchy contains one or more instances of the layer below it. The user may select a particular level from one the three list boxes along the left side of the C-ME window.

A *project* is a collection of entities created around a particular research goal, organization, or any other meaningful category that a research team might create. Each project can have an associated image to provide context for any data to be added to the project. Projects, in turn, contain *entities*, which can be either a 3-D atomic coordinate file created in Protein Data Bank format, a 2-D image file created in PNG, GIF, or JPG format, or a set of microscope images from a clinical blood sample in the case of specimen entities. 3-D entities can be rotated, zoomed and translated to orient the view as required. Entities can then be annotated, by adding notes, documents, images, e-mail threads, or other forms of information. *Annotations* are anchored to specific locations in the 2-D or 3-D entities, so an annotation is always placed in a specific context and in a precise relationship to related annotations.

The C-ME application does not provide advanced molecular viewing features, such as surface display, geometry analysis, and electron density viewing. It is not intended to replace existing advanced or specialized molecular viewers, such as Pymol or Coot [12], [13]. Instead, other viewers can be launched from C-ME to provide more detailed molecular analysis.

C-ME provides basic 2-D image analysis functionality which includes the following image manipulation functions: RGB color filtering, zooming, and basic image enhancements such as contrast and brightness control. Advanced image analysis operations should be performed with an appropriate external image manipulation application (e.g. Adobe Photoshop or a purpose-specific image analysis application).

### User Interface

**Figure 5:**
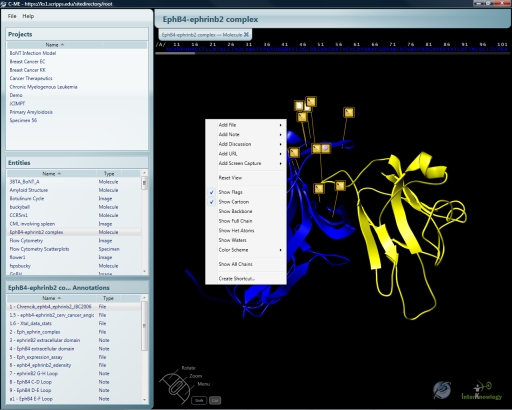
Context menu for 3D molecule entity. Right-clicking the mouse provides functionality based on the context. In this case, there are options for adding annotations, changing the molecule's rendering and creating a shortcut. The Project, Entity and Annotation windows are seen along the left side of the application window.

C-ME presents a user with an intuitive interface that reflects the three levels of information within the system. Individual windows on the left side of the screen reflect the selections made by the user at each level of the C-ME system (Figure 5). The top left window reflects the projects that are currently established on the system. The middle left window reflects the entities that have been chosen for examination by the user. The lower left window reflects the list of annotations that have already been entered by other users and allows users to navigate through them as well as to open the annotations for viewing.

Since MOSS 2007 creates a central repository for data in almost any format, users are not limited in the ways they organize their data. Being able to link multiple kinds of data to an image or a protein structure, for example, makes it easier to organize experiments, collect and distribute results, and speed up the process of uncovering deeper knowledge from various experiments. Each entity, sample, or laboratory report can become its own site. All information about that entity can be collected and retrieved from one point either using C-ME or a web browser. A movie demonstrating major C-ME features and usage is available (Video S1, Supporting Information).

### Annotations

**Figure 6:**
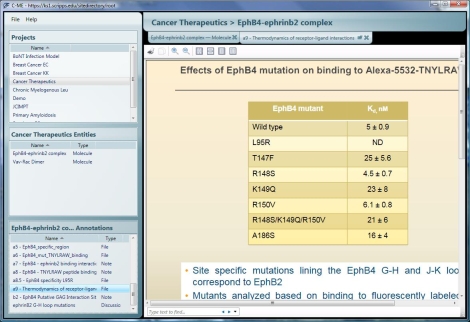
C-ME tabbed viewing environment. Supported file types are opened in a new tab to save space on the desktop. This example shows a PowerPoint presentation annotation displayed in a tab.

Once an entity is created, a user can begin annotating the molecule, image or specimen. Annotations to molecule entities can be attached to the entire molecule, a set of user-defined amino acid residues, or a set of user-defined atoms. For an image entity, annotations are attached to the entire image or to a user-selected region of the image. Specimen entities are different in that the annotations are actually the set of images (called Cell Hit annotations) associated with a specific sample added to the entity when the specimen entity was created. Additional images can be added to a specimen entity by right-clicking on the entity name in the Entities window at the left. Annotations provide additional information and points of discussion for a molecular structure. Text notes, URLs, files, screen captures and discussions are all types of annotations that can be added to a structure or image. A user can then either double-click the annotation from the list in the lower left window or select the annotation flag from the graphical window to open that annotation. Office documents and PDF files are opened for reading in a new tab in the C-ME graphical window (Figure 6). The user may then decide to open that document for editing.

### Files and Office Documents

Any file can be attached as an annotation. Commonly used file annotations include generic text files, images, audio or video media files, and PDF files. C-ME relies on the user's installed software to open these files with the appropriate application. In addition, Microsoft Word, Excel and PowerPoint documents can be attached as annotations in C-ME and are more tightly integrated with C-ME and open in a separate tab in the graphical window. For example, Excel spreadsheets can be attached as annotations and accessed through C-ME, where data or formulas in the cells can be modified by users with appropriate permissions from local or remote locations. Multiple users can open a given document simultaneously. A check-in/check-out feature is available. When a user edits an annotation, it is checked out to the first person who edits it. Another user who wants to edit the same file at the same time can only open it as a “Read-Only” version.

### Notes

General text descriptions can be added as a Note annotation and can be attached to the entire entity or to a specific region of the entity. A note annotation flag appears as a small pad-and-pen icon.

### URL

A Uniform Resource Locator (URL) annotation is an HTML link that can be attached to an entity. This allows any web page to be added as additional information without having to actually extract information from the web site.

### Screen Capture

A screen capture annotation allows an arbitrary rectangle of the user's screen to be captured as an image and attached to an entity. When selecting a screen capture annotation, C-ME is minimized and the rest of the desktop is shown with a light grey color. The mouse cursor becomes a cross-hair cursor which can be used to drag out a rectangle to save as the screen captured image. A window then appears with the captured image in the bottom pane and a place to enter a title for the screen capture annotation, and optionally, an associated URL and additional notes.

### Discussions

Users can also discuss aspects of an entity by creating a discussion thread. When creating a new discussion annotation, a window appears to enter the subject of the discussion and the actual text of the discussion. Another user can then double-click this discussion and select a particular subject and click on the Reply button to continue the discussion. Other users with access to that entity can view all the entries in the discussion and contribute if desired.

### Forms

Microsoft InfoPath forms, which are XML-based and are used to enter data about experiments and samples, can be published directly into a custom list or automatically sent via an e-mail message to a predefined e-mail account. InfoPath forms are currently being used to gather pathologist reviews of microscope images of stained cells. These forms are then processed to determine an overall score for each image from each pathologist's review. These scores can then be used to classify a cell image.

### Security

By relying on industry-standard commercial off-the-shelf software products, C-ME addresses three areas of concern related to security: authentication and information access, data backup, and data transport. C-ME is built on the Windows Server 2003 operating system, and relies on the Active Directory for authentication and access control. System administrators or lab directors can control access and grant varying levels of privilege—from “Read Only,” to “Contribute,” to “Design Pages”—or deny access entirely. The data backup function is built into the MOSS 2007 data portal and data contributed to C-ME is automatically backed up. Furthermore, data is encrypted and transferred using the SSL standard.

### C-ME Use Cases

Generally, C-ME is being used during regular laboratory operations to discuss and share current progress and problems in a particular research project. Rather than having to create new PowerPoint presentations, the presenter can start the C-ME application and browse to the appropriate project to share the current thoughts and results. The annotations placed there collaboratively by other researchers working on that project can be viewed and edited, and new annotations can be added on the spot.

Similarly, C-ME is being used to provide a tour of a completed crystal structure using the published research paper as a basis. Now, the reader can step through the C-ME annotations extracted from the paper and watch as the relevant portions of the structure are highlighted to place additional data, such as activity assays, sequence comparisons, and protein purification gels, in the context of the structural features. In addition, this guided display feature of C-ME will also be used to share ideas, concepts and data with external collaborators allowing them to review, amend or add annotations.

From the bench-top perspective, C-ME is being used in both structure-based and cell-based research projects. There are currently two primary projects which represent similar but different challenges for the collaborative environment. The Functional and Structural Proteomics analysis of SARS-CoV related proteins (FSPS, http://visp.scripps.edu/SARS/default.aspx) is an NIH-funded project that requires the production of over 30 SARS virus proteome proteins, their crystallization, and eventually, determination of their crystal structures. C-ME is being used to collect, organize and share the known FSPS structures as well as to annotate them with functional study information.

The second project is the Cancer Bioengineering Research Project (CBRP, http://cancer.scripps.edu). This project is focused on detecting rare circulating tumor cells in blood specimens from cancer patients. These cells have similarities that allow for their automated detection, and once detected, a 2D image of each cell is produced and is analyzed in multiple ways, requiring annotation of various types. These image analysis results need to be associated with the patient sample they came from, and each specimen might result in 200+ different images, each of which requires evaluation and annotation by a pathologist. C-ME is being used to store and organize the scanned microscope cell images, with the image analysis annotations and scoring attached to each stored image. In addition, InfoPath forms are used to allow pathologists to input the image analysis data to determine whether circulating tumor cells exist.

C-ME is being used in clinical classrooms. For example, the viewing and annotation features make it suitable for advanced-level molecular biology courses. Using C-ME, instructors can present molecular-based material to students at a level of detail not available in print textbooks. It also organizes course materials in hierarchical trees and makes it possible for students to experience the process of research collaboration with their instructors. An instructor in a graduate level pathology course, for example, uses C-ME to present students with examples of pathology reports in 2-D image formats. The instructor prepares appropriate annotations ahead of class time, and students reviewing the material after class can add comments or leave questions for the instructor. Additionally, image-based medical specialties can use the features of C-ME for education. In a pathology fellowship training program, where visual microscopic skills are paramount, learning modules for various pathologic diseases are created. This gives trainees the opportunity to practice making diagnoses by looking at microscopic images, as they will in their future daily practice. But unlike in real life, these microscopic images come with embedded annotations, files, PowerPoint presentations and on-line literature reviews, highlighting the corresponding disease information the trainees need to master in association with each image of abnormal cells. Trainees can also add modules to C-ME as they pursue special interests in one disease or another, eventually creating a training ‘wiki’ for future year trainees.

### Future Directions

While C-ME contains significant capability for use by researchers today, the application will be enhanced to increase its utility. Currently, C-ME requires an internet connection to access the project and entity data and annotations. We are planning enhancements that would allow offline use of C-ME with data synchronization when C-ME is re-connected to the internet.

C-ME will also be extended to leverage the search and indexing functionality already present in MOSS 2007 to provide searching functionality from the C-ME client application. A C-ME user would be able to perform searches within and across projects or entities to find relevant documents, notes, and other annotations related to the search criteria.

In addition, C-ME will detect user presence to signal a user that a collaborator is currently on-line. This would allow users to send an email or initiate an instant messenger session to discuss annotations and results if the other users or collaborators are currently using C-ME.

Currently, InfoPath Forms are used to capture pathologists' scores from surveys designed to evaluate cell images. The use of InfoPath forms will be extended to include tests and quizzes for use by instructors to evaluate student performance and understanding of material presented in a C-ME project or entity.

Collaborative Molecular Modeling Environment (C-ME) enhances existing laboratory resources with interactive, real-time annotation and visualization capabilities. With this technology, researchers can create wiki-style collaborations for internal or distributed laboratory environments. As a content management and collaboration tool, Collaborative Molecular Modeling Environment (C-ME) has the potential to improve research efficiency by providing collaborators real-time access to data organized at the molecular and project levels. Its platform consolidates data from disparate sources, allowing users to easily compare, contrast, and merge information from different file systems. It complements existing visualization and collaboration software tools, such as iSee, Kinemage, MICE, and BioCoRE by allowing for real-time two-dimensional and three-dimensional annotation, hierarchical project and data organization, and dynamic collaboration.

The C-ME installation is available for download for computers running the Windows XP or Vista operating system (http://c-me.scripps.edu). This web site also contains additional information about the C-ME application.

## Supporting Information

Video S1 Video demonstration of the C-ME application in use. The major C-ME features are shown using a 3D protein structure entity and its associated annotations as an example.
(9.82 MB SWF)
